# Testing the construct validity of hospital care quality indicators: a case study on hip replacement

**DOI:** 10.1186/s12913-016-1778-7

**Published:** 2016-10-05

**Authors:** Claudia Fischer, Hester F. Lingsma, Helen A. Anema, Job Kievit, Ewout W. Steyerberg, Niek Klazinga

**Affiliations:** 1 Department of Public Health, Centre for Medical Decision Making, Erasmus MC, Dr. Molewaterplein 50, NA-2217, 3000 CA Rotterdam, The Netherlands; 2 Department of Public Health, Amsterdam Medical Centre, Meibergdreef 9, 1105 AZ Amsterdam, The Netherlands; 3 Department of Medical Decision Making, Leiden University Medical Centre, LUMC-J10-S, PO Box 9600, 2300 RC Leiden, The Netherlands

**Keywords:** Hip replacement, Database, Health care quality, Quality indicators, Validity

## Abstract

**Background:**

Quality indicators are increasingly used to measure the quality of care and compare quality across hospitals. In the Netherlands over the past few years numerous hospital quality indicators have been developed and reported. Dutch indicators are mainly based on expert consensus and face validity and little is known about their construct validity. Therefore, we aim to study the construct validity of a set of national hospital quality indicators for hip replacements.

**Methods:**

We used the scores of 100 Dutch hospitals on national hospital quality indicators looking at care delivered over a two year period. We assessed construct validity by relating structure, process and outcome indicators using chi-square statistics, bootstrapped Spearman correlations, and independent sample t-tests. We studied indicators that are expected to associate as they measure the same clinical construct.

**Result:**

Among the 28 hypothesized correlations, three associations were significant in the direction hypothesized. Hospitals with low scores on wound infections had high scores on scheduling postoperative appointments (*p*-value = 0.001) and high scores on not transfusing homologous blood (correlation coefficient = -0.28; *p*-value = 0.05). Hospitals with high scores on scheduling complication meetings, also had high scores on providing thrombosis prophylaxis (correlation coefficient = 0.21; *p*-value = 0.04).

**Conclusion:**

Despite the face validity of hospital quality indicators for hip replacement, construct validity seems to be limited. Although the individual indicators might be valid and actionable, drawing overall conclusions based on the whole indicator set should be done carefully, as construct validity could not be established. The factors that may explain the lack of construct validity are poor data quality, no adjustment for case-mix and statistical uncertainty.

## Background

As quality improvement becomes a central tenet of health care, quality indicators (QIs) are becoming increasingly important. Quality is monitored and publicly reported in order to provide patients and health insurers with information regarding choices and to improve the quality of the underlying complex and resource-intensive care procedures [1].

For such purposes QIs need to be based on reliable data [2, 3], and they must cover quality aspects on a structural, process, and outcome level [4]. The underlying assumption is that good structures of care increase the likelihood of good processes and good processes increase the likelihood of good outcomes (the Donabedian framework) [4]. Another important prerequisite for the external use of the indicators and fair comparison of hospitals is that QIs are valid [5] and actionable. QIs need to provide insight into which factors determine the occurrence of an outcome, so that hospitals are able to act on the process to improve the outcome.

Total hip replacements are interesting for quality of care research because hip replacements are common, elective procedures that are being performed more and more frequently [6]. Although the clinical and economic effectiveness of hip replacements is proven [7], it is still possible to observe variation in performance between providers [8, 9]. As a result, these orthopaedic procedures have for instance been included in pay-for-performance schemes by social insurance programs such as Medicare and Medicaid [10]. In such a program hospitals are rewarded for meeting pre-defined performance targets related to the health care that is delivered [11]. In the pay-for-performance scheme of Medicare and Medicaid, the so-called ‘Premier Quality Initiative Demonstration’, a composite score was created from three measures of surgical process quality and three measures of surgical outcome. A performance bonus consisting of two percent of diagnosis-related group payments for total hip and knee arthroplasty was given to hospitals that scored in the top 10% on the composite measure [10]. For such external use (as well as for internal use such as in local hospital quality improvement), it is critical that indicators present a valid picture of the quality of the health care that is provided by a hospital [5]. However, empirical evaluations of the relation between outcome indicators and process and structure indicators that measure the same construct are scarce in Europe [12]. Even if quality indicators are tested in different health care systems, an evaluation in the health care system in which the indicator is used is essential. Differences in national health care and local hospital organization may influence the indicator’s validity [1]. Insight into the validity of QIs is particularly important when data reliability is at stake, for instance when there are no national standards that hospitals or database software providers should follow when setting up their in-hospital quality registries in which the quality data is entered [1, 2]. This is the case in the Netherlands, where QIs were developed by the Dutch Health Care Transparency Program (DHTP) through a combination of expert consensus and available scientific literature. They were tested in only a few hospitals. Employees of the hospitals are required to calculate and report these QIs annually to the DHTP; public reporting and publication of these QIs has occurred for several subsequent years [13].

Therefore we aimed to evaluate several publicly available indicators of quality of hospital care in the Netherlands related to hip replacements (15 indicators) with regard to their construct validity, or the “degree to which an indicator measures what it claims to be measuring” [14]. In this study construct validity is operationalized by a significant associationbetween two quality indicators that measure the same underlying construct in the expected direction.

## Methods

We conducted a cross-sectional data analysis, using quantitative data from two registration years (2008 and 2009) as reported by the hospitals.

### QIs under investigation

The QIs we evaluated are all related to pre-operative and post-operative health care for hip replacements. We used data from two consecutive years. Table 1 shows an overview of the definitions, numerators (i.e. number of patients who underwent a certain care process) and denominators (i.e. total number of patients) of the structure, process and outcome (S-P-O) QIs evaluated in this study. Moreover, it can be seen that the structure QIs in the hip replacement set are dichotomous (yes/no), whereas the majority of the process and outcome indicators are continuous measures (a proportion of patients with particular treatment or outcome).

**Table 1:** Included DHTP total hip replacement quality indicators

Total hip replacement			
Qi number^a^	Qi name	Indicator type^b^	QI definition
qi1	Preoperative patient information	S	Definition: hospitals provide written or audio-visual preoperative patient information (yes/no)
qi2a	Guideline thrombosis prophylaxis	S	Definition: hospitals have a guideline or protocol on thrombosis prophylaxis for cases of hip replacement (yes/no)
qi2b	Thrombosis prophylaxis	P	Definition and Numerator: in hip replacement cases, the number of operations in which patients received medical thrombosis prophylaxis within 6 weeks and no more than 3 months after the operation
qi3a	Complication register	S	Definition: an automated information system is available to provide insight into the occurrence of complications (e.g. wound infection, lung emboli) within 6 weeks of HR (yes/no)
qi3b	Appointment within 6 weeks	S	Definition: to detect complications, a postoperative appointment is held within 6 weeks of a hip replacement (yes/no)
qi3c	Orthopaedic registration form	S	Definition: in hip replacement cases, an orthopaedic registration form is used to register complications (yes/no)
qi3d	Complications meeting	S	Definition: minuted meetings are held to discuss hip-replacement complications (number of meetings per year)
qi3e	Improvement plan	S	Definition: minuted meetings are held to discuss hip-replacement complications, if necessary an improvement plan with the person in charge is assigned (yes/no)
qi4a	Blood management guideline	S	Definition: a blood-management guideline or protocol to reduce perioperative administered in case of hip replacement is present (yes/no)
qi4b	Transfusion of homologue blood	P	Definition and Numerator: in hip replacement cases, the number of operations in which patients did not receive transfusion of homologue blood
qi5a	Guideline for antibiotic prophylaxis	S	Definition: a guideline/protocol is available for antibiotic prophylaxis in the event of hip replacement (yes/no)
qi5b	Perioperative antibiotics	P	Definition and Numerator: in hip replacement cases, the number of operations in which perioperative antibiotics were administered
qi5c	Antibiotics 60–15 min	P	Definition and Numerator: in hip replacement cases, the number of operations in which patients received antibiotics 60 to 15 min before incision
qi5d	Wound infection	O	Definition and Numerator: in hip replacement cases, the number of patients with deep wound infections within 6 weeks of the operation
qi6	National prosthetic register	S	Definition: the hospital participates in the national arthroplasty register (yes/no)

^a^According to number in DHTP hip and knee replacement indicator set

^**b**^ *S* structure, *P* process, *O* outcome

### Data source

#### Dutch health care transparency program data (DHTP)

The QI data originate from a national database hosted by the DHTP [15]. Dutch hospital staff annually collect and submit to DHTP hospital-specific performance scores (numerators and denominators) for various diseases and interventions based on health care delivered in the preceding calendar year.

Although we had data on indicator scores for three subsequent years (2008, 2009, 2010) we only could include indicator scores from two years (2008, 2009) in our study. This is due to major changes in the indicators, which would have influenced the comparability of the indicator scores between the years. For our study we selected the available numerators and denominators for each hospital and indicator. All QI scores were aggregated on the hospital level (Table 1).

### Analysis

To describe the range in scores across hospitals we calculated the mean and interquartile range (IQR) of all indicator scores and denominators on the hospital level.

Based on the indicator manual, the literature and medical expert opinion, we hypothesized 28 associations between hip replacement indicators that measure the same underlying construct. Table 2 shows an overview of the hypothesized indicator associations and their direction of association.

**Table 2:** Hypothesized indicator association and direction of association

	Hypothesized indicator associations	Evidence for expected indicator association	Expected correlation association	*p*-value indicator	
				Association strength	
				2008	2009
ha^a^ 1	having a thrombosis prophylaxis management guideline (qi2a^b^) and the percentage of patients who accurately receive a thrombosis prophylaxis (qi2b)	[29]	positive	/	/
ha 2	having a blood management guideline (qi4a) and the percentage of patients who do not receive a blood transfusion (qi4b)	[29, 30]	positive	/	/
ha 3	having a guideline for antibiotic prophylaxis (qi5a) and the percentage of patients who receive antibiotic prophylaxis perioperative (qi5b) [25]	[29]	positive	/	/
ha 4	having a guideline for antibiotic prophylaxis (qi5a) and the percentage of patients who receive antibiotic prophylaxis 60–15 min before incision (qi5c) [25]	[29]	positive	/	/
ha 5	the percentage of patients who receive their perioperative antibiotic prophylaxis in a timely manner (qi5b) and the percentage of patients with deep wound infection (qi5d) [26–28]	[30–32]	negative	/	0.74
ha 6	the percentage of patients that receive antibiotic prophylaxis 60–15 min before incision (qi5c) and the percentage of patients with deep wound infection (qi5d) [26–28]	[30–32]	negative	0.14	0.74
ha 7	the percentage of patients who receive no blood transfusion (qi4b) and the percentage of patients with deep wound infection (qi5d) [29, 30]	[33, 34]	negative	0.05	0.07
ha 8	having a timely postoperative appointment (q3b) and the percentage of deep wound infections (qi5d)		negative	/	0.001
ha 9	having a complication register (qi3a) and providing a thrombosis prophylaxis (qi2b)		positive	0.73	0.19
ha 10	having a complication register (qi3a) and the percentage of patients receiving no blood transfusion (qi4b)		positive	0.09	0.57
ha 11	having a complication register (qi3a) and the percentage of patients receiving perioperative antibiotic prophylaxis (qi5b)		positive	/	0.60
ha 12	having a complication register (qi3a) and the percentage of patients receiving antibiotic prophylaxis 60–15 min before incision (qi5c)		positive	0.29	0.57
ha 13	having a complication register (qi3a) and the percentage of patients with deep wound infection (qi5d)		negative	0.74	0.43
ha 14	having an orthopaedic registration form (qi3c) and the percentage of patients receiving thrombosis prophylaxis (qi2b)	[35]	positive	0.80	0.89
ha 15	having an orthopaedic registration form (qi3c) and the percentage of patients receiving no blood transfusion (qi4b)	[35]	positive	0.98	0.26
ha 16	having an orthopaedic registration form (qi3c) and the percentage of patients receiving perioperative antibiotic prophylaxis (qi5b)	[35]	positive	/	0.06
ha 17	having an orthopaedic registration form (qi3c) and the percentage of patients receiving antibiotic prophylaxis 60–15 min before incision (qi5c) [31]	[35]	positive	/	0.28
ha 18	having an orthopaedic registration form (qi3c) and the percentage of patients with deep wound infections (qi5d) [31]	[35]	positive	0.60	0.42
ha 19	having complication meetings (qi3d) and the percentage of patients receiving thrombosis prophylaxis (qi2b)		positive	0.50	0.04
ha 20	having complication meetings (qi3d) and the percentage of patients receiving no blood transfusion (qi4b)		positive	0.26	0.91
ha 21	having complication meetings (qi3d) and the percentage of patients receiving perioperative antibiotic prophylaxis (qi5b)		positive	/	0.16
ha 22	having complication meetings (qi3d) and the percentage of patients receiving antibiotic prophylaxis 60–15 min before incision (qi5c)		positive	0.26	0.32
ha 23	having complication meetings (qi3d) and the percentage of patients with deep wound infections (qi5d)		negative	0.39	0.91
ha 24	having an improvement plan to avoid complications (qi3e) and the percentage of patients receiving thrombosis prophylaxis (qi2b)		positive	0.86	0.52
ha 25	having an improvement plan to avoid complications (qi3e) and the percentage of patients receiving no blood transfusion (qi4b)		positive	0.09	0.17
ha 26	having an improvement plan to avoid complications (qi3e) and the percentage of patients receiving perioperative antibiotic prophylaxis (qi5b)		positive	/	0.39
ha 27	having an improvement plan to avoid complications (qi3e) and the percentage of patients receiving antibiotic prophylaxis 60–15 min before incision (qi5c)		positive	0.51	0.05
ha 28	having an improvement plan to avoid complications (qi3e) and the percentage of patients with deep wound infections (qi5d)		negative	0.26	0.72

^a^ hypothesized association (ha), ^b^quality indicator (qi)

To initially investigate the relationship between continuous structure, process and outcome indicators, we used non-parametric Spearman correlations. To assess the uncertainty in the estimated correlation coefficient we calculated 95 % confidence intervals. To give a more robust estimation, these intervals were additionally estimated (bootstrapped) based on 1000 random replicas (fictitious hospitals) that were constructed from the original dataset. The relationships between the dichotomous structure indicators were analysed by means of chi-square tests. Finally, to examine the relationship between dichotomous structure and continuous process/outcome indicators independent sample t-tests were applied. Here we also bootstrapped 1000 random replicas. Analyses were conducted in the statistical programs SPSS version 21. Significance was set at α < 0.05. *P*-values below 0.1 were regarded as marginally significant.

## Results

On average 64 hospitals provided data to calculate indicator scores in year 2008, from a total of 100 available hospitals in the Netherlands. The participation increased in subsequent year, in which on average 95 % of the hospitals provided data. Many indicator scores improved from 2008 to 2009. For example, the percentage of wound infections ranged from 0 to 3 % across hospitals in 2008, while in 2009 the range was from 0 to 0.03 % (Table 3).

**Table 3:** Hospital-level variation in total hip replacement scores in year 2008 and 2009

		2008						2009					
			Indicator scores on hospital level			Denominators on hospital level			Indicator scores on hospital level			Denominators on hospital level	
		N^b^	mean	IQR	min-max	median	IQR	N^b^	mean	IQR	mix-max	mean	IQR
qi^a^1	preoperative patient information	68	1	1–1	1–1	/	/	97	1	1–1	0–1	/	/
qi2a	guideline thrombosis prophylaxis	68	1	1–1	1–1	/	/	68	1	1–1	1–1	/	/
qi2b	thrombosis prophylaxis	64	100	100–100	95–100	245	49–745	95	100	100–100	93–100	226	56–647
qi3a	complication register	68	1	1–1	0–1	/	/	97	1	1–1	0–1	/	/
qi3b	appointment within 6 weeks	68	1	1–1	1–1	/	/	97	1	1–1	0–1	/	/
qi3c	orthopaedic register form	68	1	1–1	0–1	/	/	97	1	1–1	0–1	/	/
qi3d	complication meeting	63	11	4–12	0–52	/	/	96	11	4–12	0–260	/	/
qi3e	improvement plan	65	1	1–1	0–1	/	/	96	1	1–1	0–1	/	/
qi4a	blood management guideline	68	1	1–1	1–1	/	/	68	1	1–1	1–1	/	/
qi4b	transfusion of homologous blood	52	91	94–100	0–100	241	49–745	90	91	88–100	11–100	222	56–647
5a	guideline for antibiotic prophylaxis	68	1	1–1	1–1	/	/	68	1	1–1	1–1	/	/
5b	perioperative antibiotics	65	100	100–100	100–100	245	49–745	65	100	100–100	100–100	226	56–647
qi5c	antibiotics 60–15 min	59	97	100–100	0–100	237	49–745	94	98	100–100	66–100	226	56–647
qi5d	wound infections	60	1	0–1	0–3	245	49–745	93	0	0–0	0–0	213	52–647
qi6	countrywide implementation	68	1	1–1	0–1	/	/	97	1	1–1	0–1	/	/
	average	64	X	X	X			95	X	X	X		

^a^Quality indicator (qi)

^b^Number of hospitals that delivered the indicator score

Based on their face validity and on the literature, we hypothesized 28 associations (hypothesized associations, ha) to be significant. We found three of these correlations to be significant in the direction hypothesized, of which one was found in the data from 2008 and two were found in the data from 2009 (ha 7, ha 8, ha 19).

As expected, hospitals that reported planning appointments within six weeks after surgery 0.01 % reported deep wound infections, compared to 0.02 % of those who did not report to plan postoperative appointments within six weeks (*p*-value = 0.001). Further, our analysis showed that hospitals with a higher percentage of patients who did not receive a homologue blood transfusion had a lower percentage of wound infections, although this correlation was only marginally significant (ha 7: *r* = -0.28, *p*-value = 0.05). Hospitals that had high scores on the number of complication meetings also had high scores on providing thrombosis prophylaxis (ha 19: *r* = 0.21, *p*-value = 0.04).

We found several indicator associations, which were not a priori expected.

We found two significant structure-structure associations. We observed that hospitals that maintained a complication registration were also more likely to score high on planning a postoperative appointment within six weeks post-surgery (χ2: 19.97, *p*-value < 0.01). Further, hospitals that reported holding complication meetings, 11 % reported to use an improvement plan compared to 0 % of those who did not report to hold complication meetings (*p*-value = 0.01). We also observed several process-process associations. Primarily, the administration of thrombosis prophylaxis correlated significantly with the administration of antibiotic prophylaxis, suggesting that hospitals that accurately administer thrombosis prophylaxis were more likely to accurately administer antibiotic prophylaxis to their patients (*r* = 0.27, *p*-value < 0.05) and, secondly, managed to do it in time (*r* = 0.28, *p*-value < 0.05).

We additionally observed a significant correlation between the administration of antibiotic prophylaxis and the administration of antibiotic prophylaxis in a timely manner (Spearman *R* = 0.46, *p*-value < 0.01).

Having an improvement plan was related to the percentage of patients who received their antibiotic prophylaxis in a timely manner; however, they were related differently than might be expected. Of hospitals having an improvement plan, 98 % reported to provide antibiotic prophylaxis, compared to 100 % of those who do not have an improvement plan (*p*-value = 0.03) (Table 4).

**Table 4:** Associations among total hip replacement indicators within the years 2008 and 2009

			quality indicator number										
			qi3a^a^	qi3b	qi3c	qi3e	qi6	qi3d	qi2b	qi4b	qi5b	qi5c	qi5d
CHI-SQUARE TEST													
Quality indicator number	Quality Indicator Name (indicator type)	Year											
qi3a	Complication register (S^b^)	2008											
		2009											
qi3b	Appointment within 6 weeks (S)	2008	\										
		2009	**19.97** (0.00)^c^										
qi3c	Orthopaedic register form (S)	2008	0.57 (0.45)	\									
		2009	0.43 (0.51)	0.09 (0.77)									
qi3e	Improvement plan (S)	2008	0.29 (0.59)	\	0.13 (0.71)								
		2009	0.28 (0.60)	4.38 (1.00)	0.13 (0.71)								
qi6	Countrywide implementation (S)	2008	1.41 (0.24)	\	0.20 (0.66)	0.10 (0.75)							
		2009	0.43 (0.51)	0.09 (0.77)	0.18 (0.67)	0.13 (0.71)							
INDEPENDENT T-TEST								SPEARMAN CORRELATION COEFFICIENT					
qi3d	Complication meeting (S)	2008	yes 11	\	yes 10.5	**yes 11**	yes 10.8						
			no 8.2 (0.41) ^d,e^		no 13 (0.81)	**no 0** **(0.01)**	no 8.0 (0.73)						
		2009	yes 11.3	yes 11.4	yes 11.7	yes 11.7	yes 11.6						
			no 13.1 (0.86)	no 12 (0.98)	no 4.5 (0.19)	no 4.7 (0.19)	no 7 (0.74)						
qi2b	Thrombosis prophylaxis (P)	2008	yes 99.9	\	yes 60	yes 99.9	yes 99.9	0.09 (0.50)^f^					
			no 100 (0.73)		no 100 (0.80)	no 100 (0.86)	no 100 (0.83)						
		2009	yes 99.9	yes 99.8	yes 99.8	yes 99.8	yes 99.8	**0.21** **(0.04)**					
			no 98.9 (0.19)	no 98.9 (0.18)	no 99.8 (0.89)	no 99.4 (0.52)	no 100 (0.65)						
qi4b	Transfusion of homologous blood (P)	2008	yes 90.7	\	yes 91	yes 99.1	yes 90.9	-0.16 (0.26)	0.08 (0.58)				
			no 98.4 (0.09)		no 90.7 (0.98)	no 100 (0.09)	no 97.4 (0.12)						
		2009	yes 91.8	yes 91.5	yes 92	yes 91.8	yes 91.5	0.01 (0.91)	0.17 (0.12)				
			no 89.3 (0.57)	no 94.1 (0.70)	no 82.9 (0.26)	no 75.8 (0.17)	no 93.6 (0.55)						
qi5b	Perioperative antibiotics (P)	2008	\	\	\	\	\	\	\	\			
		2009	yes 99.7	yes 99.6	yes 99.6	yes 99.7	yes 99.6	0.15 (0.16)	**0.27** **(0.01)**	0.15 (0.17)			
			no 99.2 (0.60)	no 100 (0.07)	no 100 (0.06)	no 97.8 (0.39)	no 100 (0.06)						
qi5c	Antibiotics 60–15 min (P)	2008	yes 97.1	\	\	yes 99	yes 97.2	0.15 (0.26)	-0.04 (0.79)	-0.11 (0.45)	\		
			no 100 ( 0.29)			no 50 (0.51)	no 100 (0.74)						
		2009	yes 98	**yes 98**	yes 98.4	**yes 98**	**yes 97.9**	0.10 (0.32)	**0.28** **(0.01)**	0.12 (0.25)	**0.46** **(0.00)**		
			no 99 (0.57)	**no 100** **(0.04)**	no 88.5 (0.28)	**no 100** **(0.03)**	**no 100** **(0.03)**						
qi5d	Wound infections (O)	2008	yes 0.8	\	yes 0.8	yes 0.84	yes 0.8	0.12 (0.39)	-0.03 (0.84)	**-0.28** **(0.05)**	\	0.20 (0.14)	
			no 1.0 (0.74)		no 1.0 (0.60)	no 0.44 (0.26)	no 0.3 (0.17)						
		2009	yes 0.01	**yes 0.01**	yes 0.01	yes 0.01	yes 0.01	0.01 (0.91)	-0.04 (0.71)	-0.19 (0.07)	-0.03 (0.74)	0.04 (0.74)	
			no 0.01 (0.43)	**no 0.02** **(0.001)**	no 0.0 (0.42)	no 0.01 (0.72)	no 0.01 (0.89)						

^a^ numbers indicate indicator numbers according to vertical indicator numbering

^b^ S = structure, P = process, O = outcome

^c^ x2 test (*p*-value)

^d^ t-test: mean group 1, 2 (*p*-value)

^e^ Interpretation: Of hospitals having an complication register, 11 % reported to hold complication meetings, compared to 8,2 % of those who do not have an complication register (*p*-value = 0.41)

^f^ Spearman correlation coefficient (*p*-value)

bold numbers indicate significance

## Discussion

By associating structure, process, and outcome indicators we measured the construct validity of national quality indicators for hip replacement. Of the 28 a priori expected associations (per year) only three were observed to be significant in the direction hypothesized. Additionally seven associations that were not a priori expected were also found to be significant. None of the associations were consistent over the two-year time period, despite the scientific foundation of the quality indicators and overall expert consensus regarding their validity. Therefore, the construct validity of the quality indicator set under evaluation seems limited. We only found three of the a priori expected associations to be significant. For example, we observed that in hospitals that scheduled an appointment with a patient within six weeks after the patient’s hip replacement, the number of relevant wound infections after hip replacement was lower compared to hospitals that did not plan such an appointment. This is consistent with the international literature and with the widely held opinion that an appointment within this period helps to detect postoperative complications at an early stage, and thereby prevent advanced severe wound infections [16]. We additionally observed several process-process associations, which in retrospect, might indicate an overall quality awareness culture on the hospital level. For example, hospitals that had high scores on the administration of perioperative antibiotics also had high scores on the administration of antibiotics prior to the incision.

Our study showed limited construct validity between the tested quality indicators. This finding is in line with existing literature. Several studies tend to show relatively weak associations between different types of quality indicators in the health care field [17–20]. Associations between quality indicators are complex and different methodological factors influence the association between them.

An important factor for construct validity is data reliability. Although the data registration showed signs of improvement in 2009 compared to 2008, data reliability remained an issue in the data of the DHTP. In previous studies it was found that differences in data collection and reporting methods used by hospital employees, such as the use of different indicator definitions, most likely influenced the comparability of the DHTP data [2]. Moreover, many of the indicators are not very specific. For instance, 9 of the 15 hip replacement indicators are dichotomous indicators (yes/no). But for example the indicator “availability of a guideline” (e.g. qi4a, qi5b), gives no information about actual adherence to the guideline.

The lack of association we found among the indicators may be explained by the limited variation and the small numbers observed among many of the included quality indicators. For example, in 2008 the average event rate for patients developing wound infections was merely 1 %. When there are few observations and event rates are that low, indicator scores will randomly fluctuate over time, even if the underlying quality of care remains constant [21].

Furthermore, an important factor influencing construct validity is the extent of case-mix correction, as case-mix factors make up a large part of observed outcome variation [22]. Lack of adjustment for patient characteristics, which are not related to quality of hospital care but influence the patients’ risk for an outcome, may lead to a biased reflection of quality of care and an unfair comparison between hospitals. As aggregated hospital-level data currently does not include information on the underlying patient characteristics, a valid and fair analysis between the hospitals cannot be guaranteed.

As quality improvement has become a central tenet of health care, QIs are becoming increasingly important. Many countries have already started their own QI program and many more are preparing to start QI programs soon. Despite the increasing number of countries implementing QI programs, the number of studies testing the validity of indicators is limited. While a number of studies have tested the construct validity of indicators in the U.S. [23–28], a limited number of such studies have been conducted in the European health care setting [12]. However, given the differences in national health care and local hospital organizations indicators should be evaluated before they are adopted from another health system. The validity of quality of care indicators cannot be assumed for a health care setting outside of the one where the indicator was developed and tested [1]. Therefore further research on the validity of the currently used indicators in the health care setting in which they are used is warranted. Several methodological lessons can be learned from our observations. In order for a QI to be valid, it must be reliable [2]. An indicator’s reliability is determined by the accuracy of the underlying data and the unambiguousness definition of the indicator [2]. Moreover, when hospital employees are responsible for collecting the data and computing the QIs, there needs to be some central control over these processes. Furthermore, to increase data reliability the software market should be regulated and standards should be set for the development of automatic data extraction software. In order to find relationships between indicators it is crucial to take into account the influence of low event rates and case-mix differences. Failing to adjust for these factors may confound the relationship between quality indicators.

Currently there is no gold standard on how to measure quality of care. We operationalized construct validity by the association between two test scores. Usually, in psychometric research, a person’s score on for example a new psychological test is associated with a score on a more established test measuring the same underlying construct [14]. In our study both test scores were derived from the same database and were both the subject of study. Merely the presence of a significant association that was expected based on the literature was considered to be a sign of construct validity of both indicators. One could argue therefore that the method of validity assessment in our study is not very strong. A better way to assess the construct validity is to relate the indicator scores of interest with measures derived from other clinical databases. However, for countries in which reliable health care databases are scarce ours is the only approach possible. Second, the judgement on the construct validity of an indicator is always arbitrary. In our study we used a significant association in the expected direction as an indication of construct validity; however, most of the significant associations were weak. Third, when assessing multiple associations one typically corrects for multiple testing, for instance with a Bonferoni correction. As we a priori planned our associations based on the available scientific evidence, we did not correct for multiple testing. However, we do realize that we have to treat the observed significant associations with caution. Further research and trend data is needed to test construct validity over a longer time period in order to be able to identify systematic indicator associations.

## Conclusion

Overall it can be concluded that despite the face validity of hospital quality indicators for hip replacement, construct validity seems to be limited. Although the individual indicators might be valid and actionable, drawing overall conclusions based on the whole indicator set should be done with caution, as construct validity could not be established. Limitations of the quality indicators that likely explain the lack of construct validity are poor data quality, lack of adjustment for case-mix and statistical uncertainty. Before any action can be taken based on the indicator scores these limitations must be addressed.
